# Erratum: Modulation of mRNA and lncRNA expression dynamics by the Set2–Rpd3S pathway

**DOI:** 10.1038/ncomms16122

**Published:** 2017-11-29

**Authors:** Ji Hyun Kim, Bo Bae Lee, Young Mi Oh, Chenchen Zhu, Lars M. Steinmetz, Yookyeong Lee, Wan Kyu Kim, Sung Bae Lee, Stephen Buratowski, TaeSoo Kim

Nature Communications 7: Article number: 13534 ; DOI: 10.1038/ncomms13534 (2016); Published 11 28 2016; Updated 11 29 2017

The ArrayExpress accession code is incorrect in this Article. The correct accession code is E-MTAB-4268.

