# Supplementary material for: Modulation of mRNA and lncRNA expression dynamics by the Set2–Rpd3S pathway
Source: Nat Commun. 2016 Nov 28;7:13534. doi: 10.1038/ncomms13534 (PMC5133700; doi:10.1038/ncomms13534)
Supplement: Supplementary Information — Supplementary Figures 1-5, Supplementary Tables 1-2 and Supplementary References. [file ncomms13534-s1.pdf]

Supplementary Figure-1

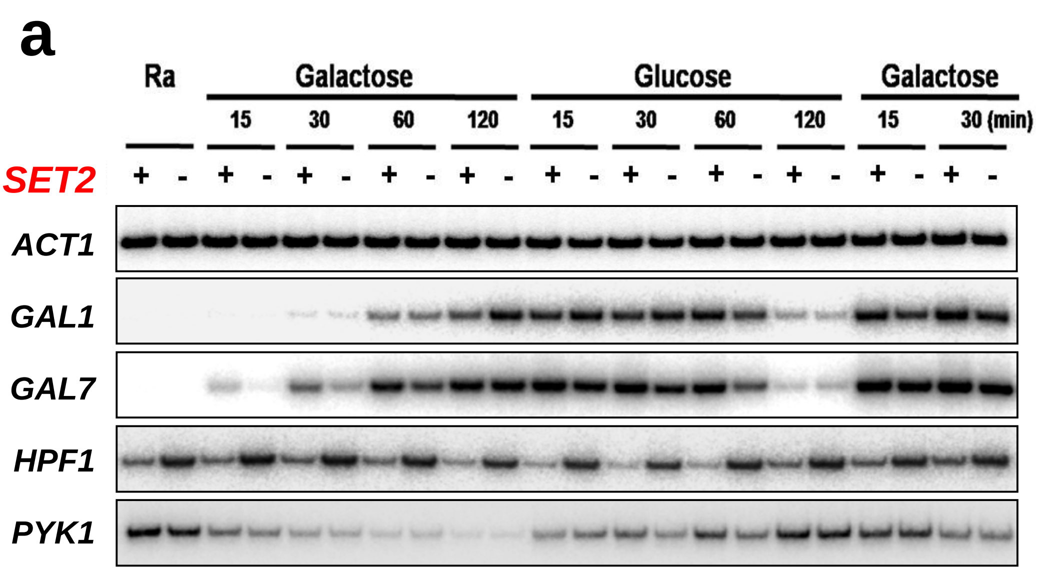

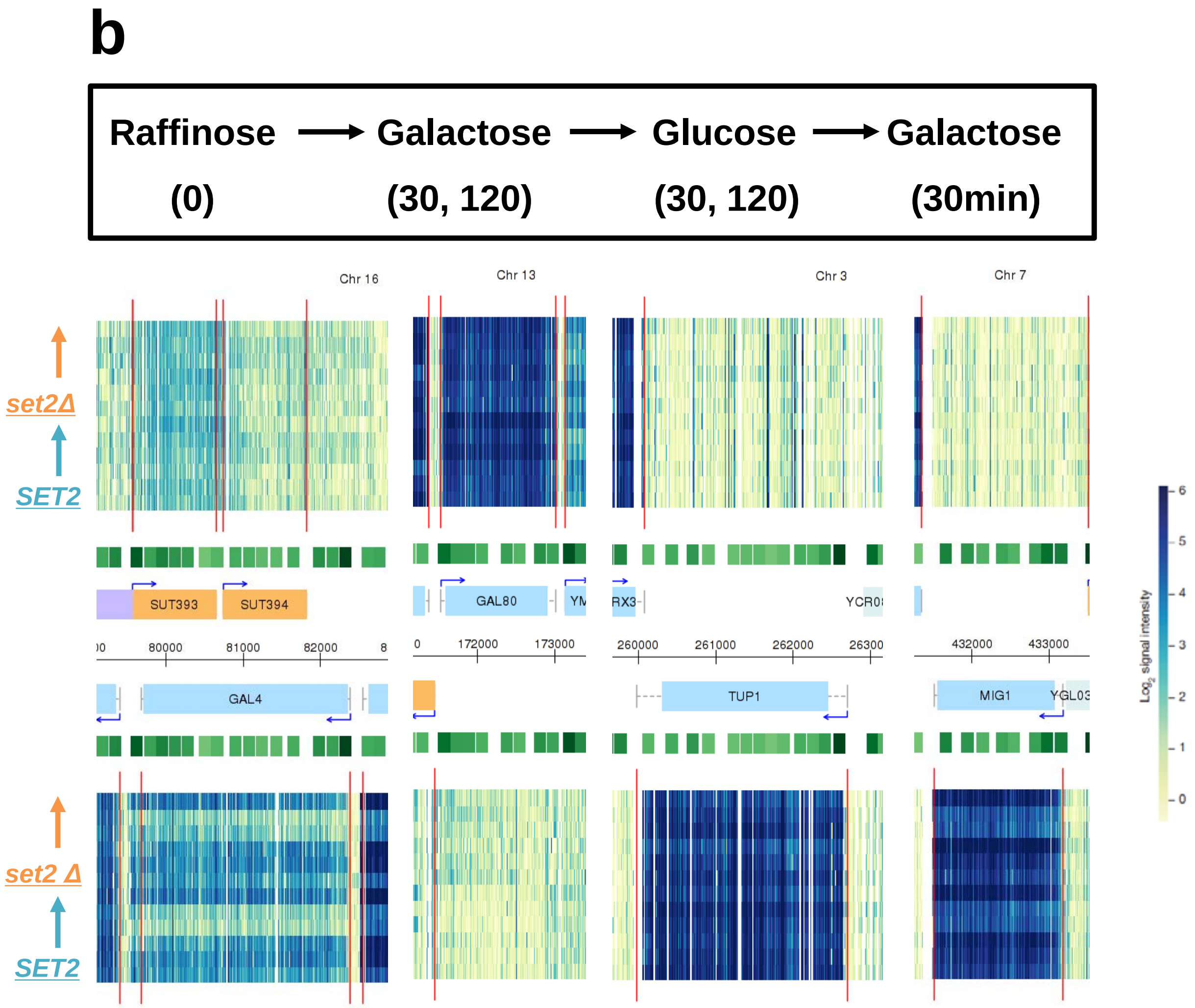

Supplementary Fig. 1

(A) Set2 negatively or positively affects gene induction. Wild type (+) and *set2*Δ (-) cells were grown in SC-raffinose media and then shifted to media containing the indicated carbon sources. Transcript levels of the indicated genes were measured by RT-PCR.

(B) Expression data of genes encoding transcriptional regulators. Expression levels are shown along the indicated gene (x axis). Normalized signal intensities (higher in dark) are shown for all time courses of *SET2* and *set2*Δ (y axis). Upper panel shows schematic representation of the time course experiments to determine changes in transcript levels upon carbon source shifts. In the lower panel, the time course is arrayed from bottom to top for the indicated strains.

Supplementary Figure-2

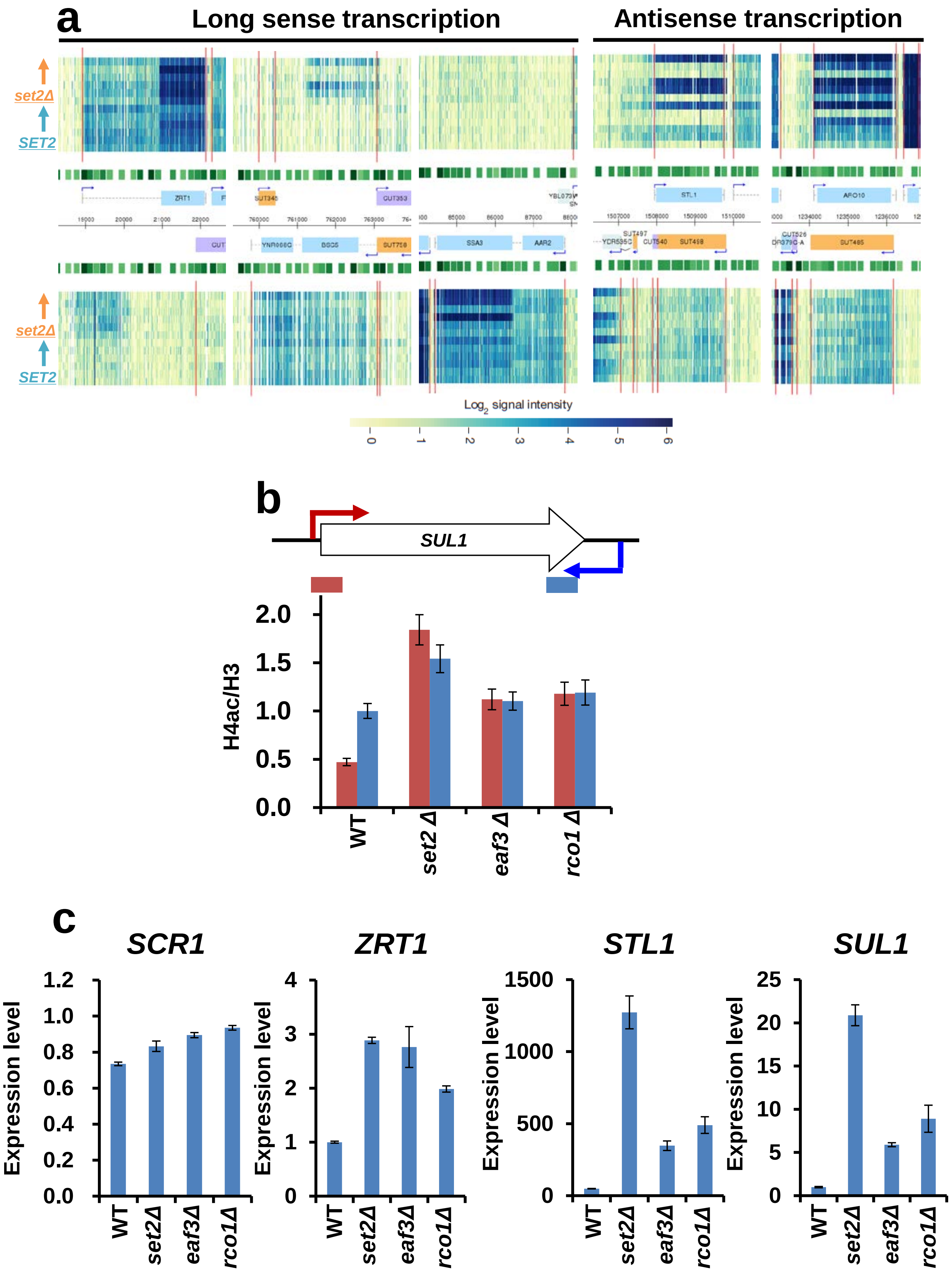

Supplementary Fig. 2

(A) Expression data of Set2-repressed genes overlapped by lncRNA transcription either from upstream or antisense promoter.

(B) Histone acetylation pattern at *SUL1* gene. ChIP analysis was done as in Figure 2d. Error bars show the standard deviation.

(C) Set2-Rpd3S negatively regulates *ZRT1*, *STL1*, and *SUL1* genes. Cells were grown in SC-raffinose media and then shifted to media containing galactose for 120 minutes. Transcript levels of the indicated genes were measured by realtime qPCR. Error bars show the standard deviation.

Supplementary Figure-3

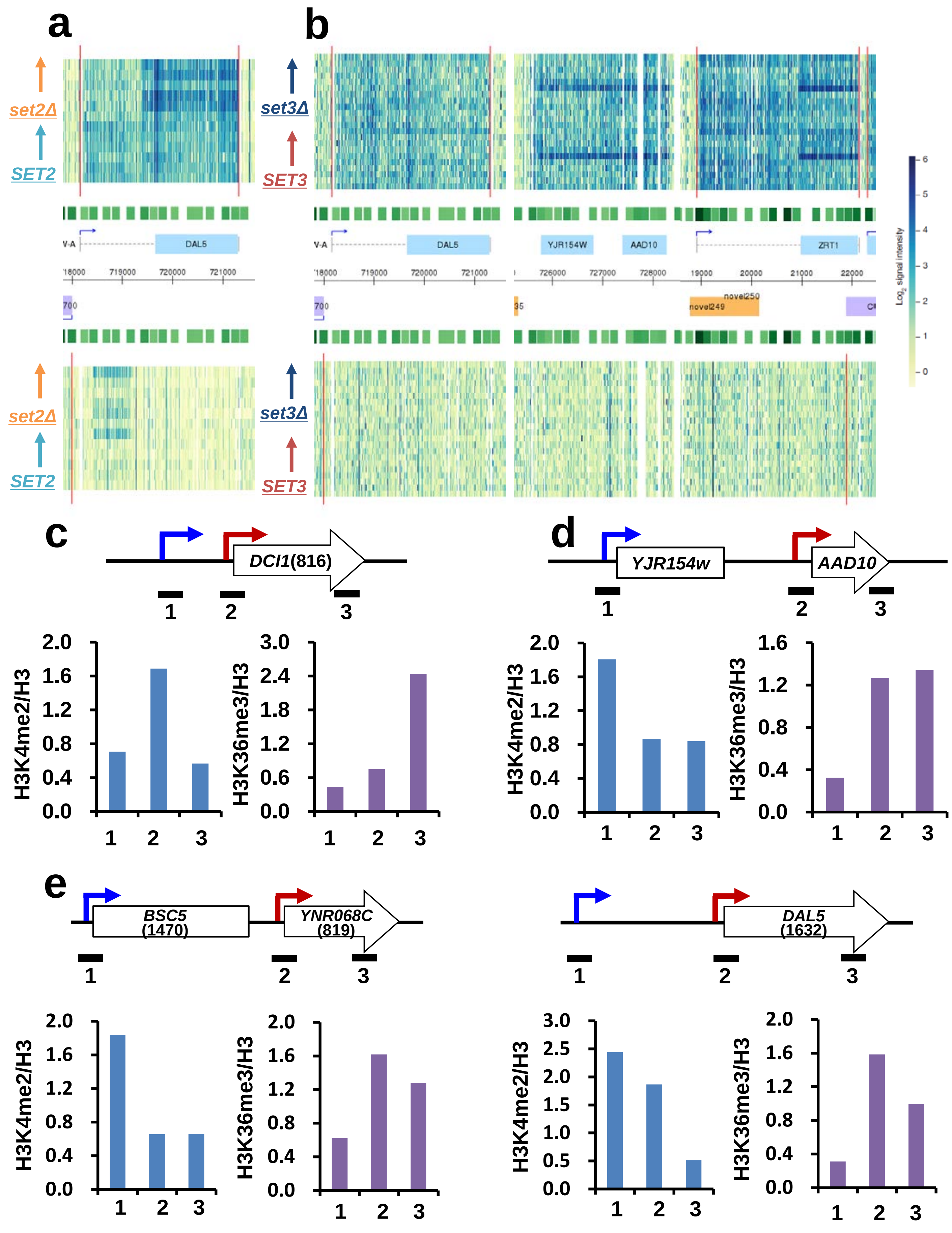

Supplementary Fig. 3

(A) Microarray data of *DAL5* response to *SET2* deletion.

(B) Set3 does not affect transcript levels of Set2-repressed genes. Microarray data from *SET3* and *set3Δ* cells [1] are shown for *DAL5* (compare to **Fig. 3a**), *AAD10* (compare to **Fig. 3b**), and *ZRT1* (compare to **Fig. 2 - Supplementary Fig. 1a**).

(C)-(E). H3K36me3 at Set2-repressed promoters. ChIP analysis for H3K4me2 and H3K36me3 was determined by Pokholok et al. [2] for cells grown in YPD. Probes are shown as black bars for the indicated genes, each of which has a long upstream transcript overlapping the mRNA promoter.

Supplementary Figure-4

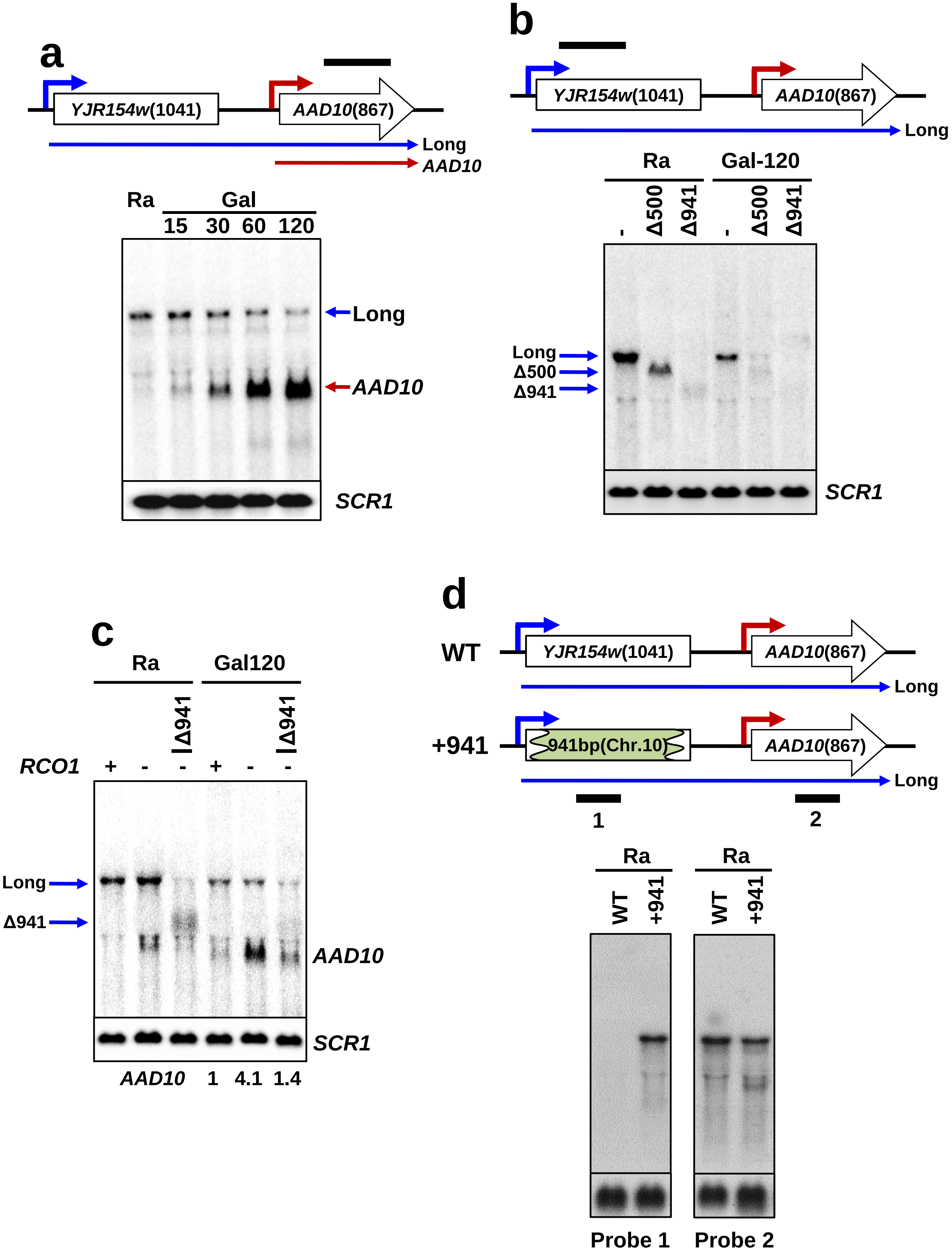

Supplementary Fig. 4

(A) Time course of raffinose (Ra) to galactose (Gal) shift. RNA was prepared and *AAD10* transcripts were analyzed by northern blot. *SCR1* was probed as a loading control.

(B) Northern blot analysis using a probe specific for the upstream RNA. Samples were analyzed exactly as in **Fig. 4b**, but the probe was at the position indicated by the black bar. This allows visualization of the upstream RNA only.

(C) Rpd3S represses *AAD10* transcription in wild type but not in Δ941 cells. Northern blot analysis of *AAD10* was done as in panel with the indicated strains. *SCR1* is used as a loading control (bottom).

(D) Northern blot analysis using WT and +941 cells grown in raffinose. Probe 1 detected only the lncRNA containing the sequence from chromosome 10 but probe 2 hybridized with both lncRNAs from WT and +941 cells.

Supplementary Figure-5

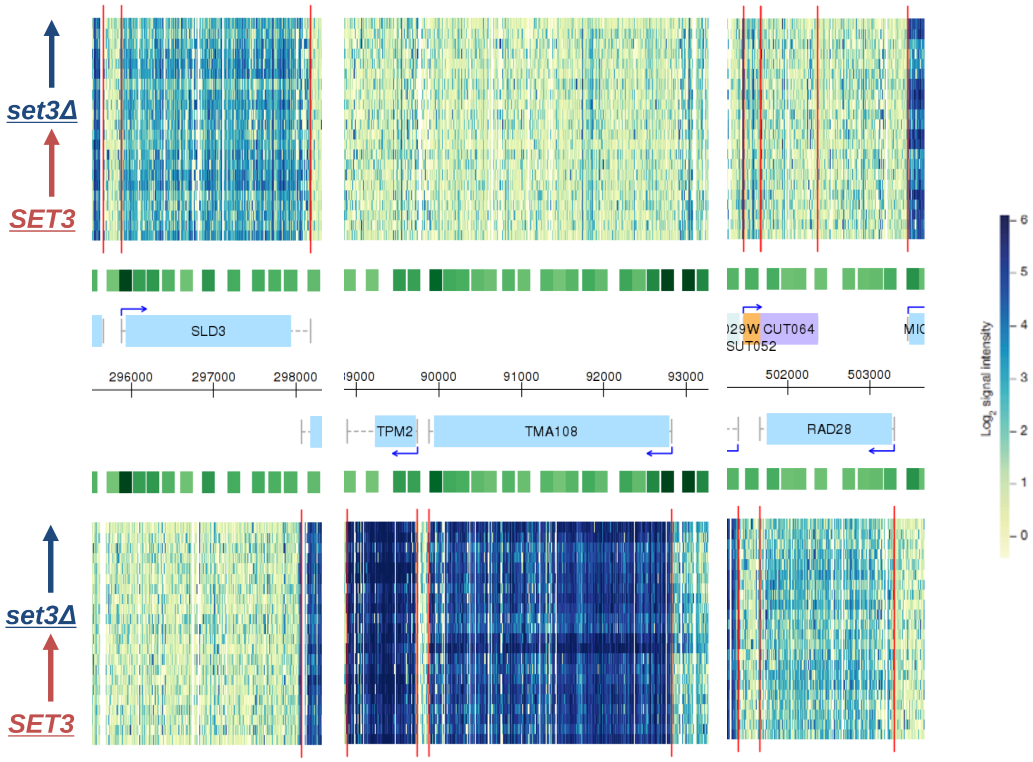

Supplementary Fig. 5

Set3 does not affect transcript levels of Set2-repressed genes. Microarray data from *SET3* and *set3Δ* cells [1] are shown for the three genes shown in **Fig. 6b**.

Supplementary Table 1 - List of Strains Used

Supplementary Table 1 includes list of yeast strains used in this study.

Strain	Genotype	Source or Reference
YSB787	MATa, bur1Δ::HIS3, ura3-52, leu2Δ1, trp1Δ63, his3Δ200, lys2Δ202 (*pRS316-BUR1*)	S. Buratowski
YSB788	MATα, bur1Δ::HIS3, ura3-52, leu2Δ1, trp1Δ63, his3Δ200, lys2Δ202 (*pRS316-BUR1*)	S. Buratowski
YSB1002	MATa, bur1Δ::HIS3, ura3-52 or ura3Δ0, leu2Δ1 or leu2Δ0, trp1Δ63, his3Δ200 or his3Δ1, lys2Δ202 or LYS2, met15Δ0 or MET15, set2Δ::KanMX (*pRS316-BUR1*)	S. Buratowski
YSB1227	MATα, bur1Δ::HIS3, ura3-52 or ura3Δ0, leu2Δ1 or leu2Δ0, trp1Δ63, his3Δ200 or his3Δ1, lys2Δ202 or LYS2, met15Δ0 or MET15, eaf3Δ::KanMX (*pRS316-BUR1*)	S. Buratowski
YSB1849	MATa, bur1Δ::HIS3, ura3-52 or ura3Δ0, leu2Δ1 or leu2Δ0, trp1Δ63, his3Δ200 or his3Δ1, lys2Δ202 or LYS2, met15Δ0 or MET15, rco1Δ::KanMX (*pRS316-BUR1*)	S. Buratowski
YSB1318	MATα, bur1Δ::HIS3, ura3-52 or ura3Δ0, leu2Δ1 or leu2Δ0, trp1Δ63, his3Δ200 or his3Δ1, lys2Δ202 or LYS2, met15Δ0 or MET15, pho23Δ::KanMX (*pRS316-BUR1*)	S. Buratowski
YSB1320	MATα, bur1Δ::HIS3, ura3-52 or ura3Δ0, leu2Δ1 or leu2Δ0, trp1Δ63, his3Δ200 or his3Δ1, lys2Δ202 or LYS2, met15Δ0 or MET15, dep1Δ::KanMX (*pRS316-BUR1*)	S. Buratowski
YSB1064	MATa, bur1Δ::HIS3, ura3-52 or ura3Δ0, leu2Δ1 or leu2Δ0, trp1Δ63, his3Δ200 or his3Δ1, lys2Δ202 or LYS2, met15Δ0 or MET15, set3Δ::KanMX (*pRS316-BUR1*)	S. Buratowski
YSB1063	MATα, bur1Δ::HIS3, ura3-52 or ura3Δ0, leu2Δ1 or leu2Δ0, trp1Δ63, his3Δ200 or his3Δ1, lys2Δ202 or LYS2, met15Δ0 or MET15, set3Δ::KanMX (*pRS316-BUR1*)	S. Buratowski
YSB1850	MATa, bur1Δ::HIS3, ura3-52 or ura3Δ0, leu2Δ1 or leu2Δ0, trp1Δ63, his3Δ200 or his3Δ1, lys2Δ202 or LYS2, met15Δ0 or MET15, rpd3Δ::KanMX (*pRS316-BUR1*)	S. Buratowski
YF336	MATa, ura3Δ0, leu2Δ0, his3Δ1, met15Δ0	Saccharomyces Genome Deletion Project

YSB2286	MATa, ura3Δ0, leu2Δ0, his3Δ1, met15Δ0, set2Δ::NATMX	S. Buratowski
YTK11	MATa, ura3Δ0, leu2Δ0, his3Δ1, met15Δ0, set2Δ::NATMX, pho23Δ::KanMX	This study
YTK13	MATa, ura3Δ0, leu2Δ0, his3Δ1, met15Δ0, set2Δ::NATMX, dep1Δ::KanMX	This study
YTK30	MATa, ura3Δ0, leu2Δ0, his3Δ1, met15Δ0, YJR154W 500bp deletion	This study
YTK31	MATa, ura3Δ0, leu2Δ0, his3Δ1, met15Δ0, YJR154W 941bp deletion	This study
YTK36	MATa, ura3Δ0, leu2Δ0, his3Δ1, met15Δ0, YJR154W 500bp deletion, set3Δ::KanMX	This study
YTK37	MATa, ura3Δ0, leu2Δ0, his3Δ1, met15Δ0, YJR154W 941bp deletion, set3Δ::KanMX	This study
YTK38	MATa, ura3Δ0, leu2Δ0, his3Δ1, met15Δ0, YJR154W 500bp deletion, set2Δ::KanMX	This study
YTK39	MATa, ura3Δ0, leu2Δ0, his3Δ1, met15Δ0, YJR154W 941bp deletion, set2Δ::KanMX	This study
YTK40	MATa, ura3Δ0, leu2Δ0, his3Δ1, met15Δ0, set3Δ::KanMX	This study
YTK41	MATa, ura3Δ0, leu2Δ0, his3Δ1, met15Δ0, set2Δ::KanMX	This study
YTK72	MATa, ura3Δ0, leu2Δ0, his3Δ1, met15Δ0, YJR154W 941bp deletion, hst1Δ::KanMX	This study
YTK73	MATa, ura3Δ0, leu2Δ0, his3Δ1, met15Δ0, hst1Δ::KanMX	This study
YTK76	MATa, ura3Δ0, leu2Δ0, his3Δ1, met15Δ0, YJR154W 941bp deletion, rco1Δ::KanMX	This study
YTK77	MATa, ura3Δ0, leu2Δ0, his3Δ1, met15Δ0, rco1Δ::KanMX	This study
YTK250	YTK31 – Insertion of a 941bp DNA from chromosome 10	This study
YTK255	YTK250, set2Δ::KanMX	This study

Supplementary Table 2 - List of Oligonucleotides Used

Supplementary Table 2 includes list of oligonucleotides used in this study.

**Gene**	**Sequences**
AAD10(prom)	up TTTTGAGCTACTTGATGCTTTTT<br>low TACATCATACCCCTTATAATCC

AAD10(northern probe)	up GACGGATATGGAGGTTAAAAT<br>low CACAATAAAAGATGCCTGCA
PYK1	up GGTAAGATCTGTTCCCACAAGGGTG<br>low CAAGTCACCTCTGGCAACCATAACAC
SAM1	up TGTCCACGAG GAGAAGGATT<br>low TCTCTTCTCG CGTCAGCCA
TEL VI	up TTTCGATCAAACTGCGTCAGG<br>low CGAGACAACGACAGGAAATAC
GAL1	up GAAGAGTCTCTCGCCAATAAGAAACAGG<br>low GAACATTCGTAAAGTTTATCGCAAG
ACT1	up TCCTTCTGTTTTGGGTTTGGAATC<br>low CCAATCCAGACGGAGTACTTTCTTTC
GAL7	up AGCCCCAATCATAATCTAACCAT<br>low TGACGTAATCGGCAAACAAAT
HGH1	up TGCGGATATCTCGAGGTTTAA<br>low TTTTTAATTGTGGAAGCGACAC
YJR154W(prom-ChIP)	up TCAGAGTCTAACATTAAGGTCA<br>low CATCGTATGGATTGCGTTTTG
YJR154W(prom-northern)	up TCAGAGTCTAACATTAAGGTCA<br>low CAGTTGAATATCACTAGTGCATT
SUL1	up TTATCGTTCTGGTGTTAGAACA<br>low AAATGACCCAGTTGCTGGAT
SUL1(prom)	up GCAGAATACTCGGAAAGAATAT<br>low CACCATTGTGAAGTCCGTCT

SUL1(3'end)	up AGCCTGGTTGATCTGAGAAAA<br>low AATAGAGTCGTCACTATATTCC
HPF1	up TCACATTCTCTCCCTACTCTAAC<br>low ACCTTCACCCTTGAATACAACAG
SCR1	up GAAGTGTCCCGGCTATAATA AA<br>low GACGCTGGATAAAACTCCCC
ZRT1	up GGGTTGAAAGAA AGTATGGTC<br>low ATACTCTACTCCGTTCTTGGT
STL1	up CCAAATGATGAGGAAGTTATAAC<br>low AAATTGCGTTGAAGCTGCAATC
DCI1	up CGGTTTTGTCGCAGAAGTGGGAAC<br>low AAGGTTCCGTTAGTCTCCATTGC
YNR068C	up GAACACAAAAGATTGCAAAC<br>low TGAACTGACCTGATTCCATT
DAL5	up CACTGCTCAATACTGGAAGA<br>low GTATCTTCTTATTGGCTGCA
PCA1	up TGGAGTTCATATCAATGAGGGAA<br>low CATAACGCAGAAGTATAGCTACA
YJR154W/pCORE	up AACTCCGGTATAGTCTACAAGGTTGGCGCTGGTGCAAGTGACCAGGGCTA GAGCTCGTTTTCGACACTGG<br>low AACGTTCACATGCTTGATGGGTCGTATGATGAACAATATCTTCTCGGTGG TCCTTACCATTAAGTTGATC
YJR154w-941 deletion	ATGAACACAGATTCACACAACCTTAGTGAGCCATACAATATAGGTGGCCAAGGGGTACTATGGAGAGACAATAAG GGTGAATTATGGGTCCACGCAATAA
YJR154w-500deletion	AGAGACCTCCAGATGTGATGAAATATTGAAAGAAATCGAACCGCATTTTTGCAGCCAGTGCAAATCGTACGTCAC AAGACAGAGTTGCTGGATATTTTTT

YJR154W(941Δ)/PCORE	up ATGAACACAGATTCACACAACCTTAGTGAGCCATACAATATAGGTGGCCAGAGCTCGTTTTCGACACTGG<br>low TTATTGCGTGGACCCATAATTCACCCTTATTGTCTCTCCATAGTACCCCTTCCTTACCATTAAGTTGATC
YJR154W/+941bp	up ATGAACACAGATTCACACAACCTTAGTGAGCCATACAATATAGGTGGCCACTTTGACTGCGCAATTGTTC<br>low TTATTGCGTGGACCCATAATTCACCCTTATTGTCTCTCCATAGTACCCCTTGTCTAAAATTCAGTATAAC
Chr10 *(+941bp-northern probe)*	up TTTCCGCTGTGCAACTAAGT<br>low GAGCTATGAAGTATACTGTTTGG

Supplementary References

1. Kim, T., Xu, Z., Clauder-Munster, S., Steinmetz, L.M. & Buratowski, S. Set3 HDAC mediates effects of overlapping noncoding transcription on gene induction kinetics. *Cell* **150**, 1158-1169 (2012).
2. Pokholok, D.K. *et al.* Genome-wide map of nucleosome acetylation and methylation in yeast. *Cell* **122**, 517-527 (2005).
